# Data Mining for Biomedicine and Healthcare

**DOI:** 10.1155/2017/7107629

**Published:** 2017-08-20

**Authors:** Zhengxing Huang, Jose M. Juarez, Xiang Li

**Affiliations:** ^1^College of Biomedical Engineering and Instrument Science, Zhejiang University, Hangzhou, China; ^2^University of Murcia, Murcia, Spain; ^3^IBM Research, Beijing, China

## 1. Introduction

Artificial intelligence (AI), as a computational method, attempts to mimic, in a very simplistic way, the human cognition capability so as to solve problems that have defined solution using conventional computational techniques. Medicine is unquestionably the main area in which AI tools and techniques have been applied. Medicine plays an essential role in human life to assess and solve problems with guarantees of success. Medicine can be considered a multidisciplinary science by itself, laying on the advances and discoveries of other sciences (biology, physics, statistics, etc.) and using such results as tools to solve diagnosis or prognosis problems. For this reason, AI in medicine has become a great issue. In this regard, developing and applying novel AI techniques and tools, due to their particular strengths, to solve medical problems and provide valuable information might be of interest to healthcare professionals in their decision making [1, 2].

AI-based medical applications can be classified into either knowledge intensive or data driven. The knowledge intensive approaches intend to obtain computational models, extracted from the clinical literature and experts' experience, to represent concepts, their relations, and the mechanisms to enable automatic reasoning to support medical decisions. Unlike knowledge intensive, data-driven approaches, at the center of the vision of learning health systems, extract such knowledge directly from the collected clinical data and this knowledge is traditionally used to explain a patient's current symptoms/vital signs and to predict future disease progression of the patient.

While an increasing amount of data is being produced by various biomedical and healthcare systems, they have not yet fully capitalized on the transformative opportunities that these data provide. Applying data-driven techniques to big health data can be of great benefit in the biomedical and healthcare domain, allowing identification and extraction of relevant information and reducing the time spent by biomedical and healthcare professionals and researchers who are trying to find meaningful patterns and new threads of knowledge.

The major goal of this special issue is to bring together the researchers in healthcare and data mining to illustrate pressing needs, demonstrate challenging research issues, and showcase the state-of-the-art research and development. The selected papers underwent a rigorous extra refereeing and revision process. It is glad to see that the selected papers presented novel methods that were empirically evaluated on medical datasets. In addition, most of the papers in this special issue include healthcare experts as coauthors, which is beneficial to open new approaches to medical experts in this multidisciplinary field. Moreover, the relevance of this special issue is strengthened by the fact that data-driven medicine is also an object of research in different communities. These communities include AI, medicine, medical informatics, decision support, and healthcare management.

## 2. The Special Issue

Medical activities can be divided into six tasks: screening, diagnosis, treatment, prognosis, monitoring, and management. Among these, diagnosis is a particular important task that data-driven approaches have been widely applied to tackle with. Diagnosis is the process of determining which disease or condition explains the patient's symptoms. The information required for diagnosis is typically collected from a patient's medical records. The paper presented by F. Yang et al. studied a problem of esophageal cancer diagnosis. Esophageal cancer is one of the fastest rising types of cancers in China, and the Kazak nationality is the highest-risk group in Xinjiang, China. They propose an effective computer-aided diagnostic system to assist physicians in interpreting digital X-ray image features and improving the quality of diagnosis. The modules of the proposed system include image preprocessing, feature extraction and selection, and image classification for disease diagnosis. They evaluated their system using 300 original esophageal X-ray images, and the experimental results show that their system is promising for the diagnostics of esophageal cancer.

As an irremediable neurodegenerative disorder that causes dementia in elderly people around the globe, Alzheimer's disease has been widely studied in medical informatics. Earlier, the majority of studies were accomplished manually or semimanually for measuring the a priori region of interest (ROI) of patient images, which are not practicable in hospital. To this end, D. Jha et al. presented an automated approach for Alzheimer's disease diagnosis. Specifically, a dual-tree complex wavelet transform was first adopted for extracting features from magnetic resonance images. Then, the authors applied principal component analysis for feature dimensionality reduction. The reduced feature vector was sent to feed-forward neural network to distinguish Alzheimer's disease and healthy control from the input MR images. The experimental results showed their approach achieved better performance than the state-of-the-art algorithms.

Reusing the data from healthcare information systems can effectively facilitate clinical trials; however, the selection of eligible patients for clinical trial recruitment criteria is still an open problem. In this issue, Y. Zhang et al. presented a computer-aided clinical trial recruitment methodology, based on syntax translation between different domain-specific languages. In their proposal, the clinical trial recruitment criteria are formally represented as general rule that are translated into intermediate query-oriented domain specific languages to map the native database queries. This approach directly uses the underlying database schema as a reference model. In consequence, the system complexity and data-mapping efforts are greatly reduced.

F. Gräßer et al. presented a recommender system for data-driven therapy decision support. They proposed two methods for therapy recommendation, namely, collaborative recommender and demographic-based recommender. Both algorithms aim to predict the individual response to different therapy options using patient data. The authors evaluated their methods using a clinical database incorporating patients suffering from the autoimmune skin disease psoriasis and showed that their methods profit from a combination into an overall therapy recommender system.

A major challenge in medical informatics is to build a regional health information exchange system. J. Lei et al. have faced this challenge in the context of health national reform in China. That is, they study the impact of the large-scale construction of hospitals and the rapid development of health information systems. Specifically, the authors used interviews, focus groups, a filed study, and a literature review to collect insights and analyze data. The case study was conducted in Xinjin region, which was able to build a complete, unified, and shared information system and many electronic health record components to integrate and manage health resources for 198 health institutions in its jurisdiction. Costs and benefits were carefully discussed, and the unique characteristics of the Xinjin case and a comparison with US cases were analyzed.
